# Whole‐transcriptome sequencing identifies neuroinflammation, metabolism and blood–brain barrier related processes in the hippocampus of aged mice during perioperative period

**DOI:** 10.1111/cns.13901

**Published:** 2022-07-27

**Authors:** Zizheng Suo, Jing Yang, Bowen Zhou, Yinyin Qu, Wenjie Xu, Min Li, Ting Xiao, Hui Zheng, Cheng Ni

**Affiliations:** ^1^ Department of Anesthesiology, National Cancer Center/National Clinical Research Center for Cancer/Cancer Hospital Chinese Academy of Medical Sciences and Peking Union Medical College Beijing China; ^2^ Department of Anesthesiology Peking University Third Hospital Beijing China; ^3^ State Key Laboratory of Molecular Oncology, Department of Etiology and Carcinogenesis, National Cancer Center/National Clinical Research Center for Cancer/Cancer Hospital Chinese Academy of Medical Sciences and Peking Union Medical College Beijing China

**Keywords:** aging, neuroinflammation, perioperative neurocognitive disorders, postoperative cognitive dysfunction, surgery and anesthesia, whole‐transcriptome sequencing

## Abstract

**Aim:**

Perioperative neurocognitive disorders (PND) occur frequently after surgery and anesthesia, especially in aged patients. Previous studies have shown multiple PND related mechanisms in the hippocampus; however, their relationships remain unclear. Meanwhile, the perioperative neuropathological processes are sophisticated and changeable, single period study could not reveal the accurate mechanisms. Thus, multiperiod whole‐transcriptome study is necessary to elucidate the gene expression patterns during perioperative period.

**Methods:**

Aged C57BL/6 mice were subjected to exploratory laparotomy under sevoflurane anesthesia. Whole‐transcriptome sequencing (RNA‐seq analysis) was performed on the hippocampi from control condition (Con), 30 min (Day0), 2 days (Day2), and 7 days (Day7) after surgery. Gene Ontology/Kyoto Encyclopedia of Genes and Genomes analyses, quantitative real‐time PCR, immunofluorescence, and fear conditioning test were also performed to elucidate the pathological processes and modulation networks during the period.

**Results:**

Through RNA‐seq analysis, 328, 3597, and 4179 differentially expressed genes (DEGs) were screened out in intraoperative period (Day0 vs. Con), early postoperative period (Day2 vs. Day0), and late postoperative period (Day7 vs. Day2). The involved GO biological processes were divided into 9 categories, and positive‐regulated processes were more than negative‐regulated ones. Seventy‐four transcription factors were highlighted. The potential synaptic and neuroinflammatory pathways were constructed for Neurotransmitter, Synapse and Neuronal alteration categories with 9 genes (*Htr1a*, *Rims1*, and *Ezh2*, etc.). The metabolic and mitochondrial pathways were constructed for metabolism, oxidative stress, and biological rhythm categories with 9 genes (*Gpld1*, *Sirt1*, and *Cry2*, etc.). The blood–brain barrier and neurotoxicity related pathways were constructed for blood–brain barrier, neurotoxicity, and cognitive function categories with 10 genes (*Mmp*2, *Itpr1*, and *Nrf1*, etc.).

**Conclusion:**

The results revealed gene expression patterns and modulation networks in the aged hippocampus during perioperative period, which provide insights into overall mechanisms and potential therapeutic targets for prevention and treatment of perioperative central nervous system diseases, such as PND, from the genetic level.

## Graphical abstract

Whole‐transcriptome sequencing reveals gene expression patterns in the aged hippocampus during perioperative period. The involved pathological processes include neuroinflammation, metabolism, and blood–brain barrier alteration, neurotoxicity, etc., which have changeable status and exert distinct roles during intraoperative, early and late postoperative periods. These results provide insights for the major mechanisms and therapeutic targets of perioperative neurocognitive disorders.

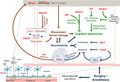

## 1 INTRODUCTION

Sixty‐six million patients over 65 years old worldwide undergo surgeries each year, including 8.5‐million Alzheimer's disease (AD) patients.^1^ Up to 40% of these patients suffered from neurological complications, termed as perioperative neurocognitive disorders (PND). PND includes postoperative cognitive dysfunction (POCD), postoperative delirium (POD), etc.^2^ Central nervous system (CNS) senescence and degeneration are the basis for PND,^3^ multiple perioperative factors including surgery and anesthesia are risk factors,^4^ and patients suffered from PND are at risk for poor functional recovery and increased mortality.^5^

Surgical trauma could trigger an acute systemic inflammation, which leads to hippocampal neuroinflammation, synaptic dysfunction, and cognitive dysfunction.^6^ Perioperative CNS pathological processes also included blood–brain barrier (BBB) damage,^7^ oxidative stress,^8^ etc. These processes have interactions during perioperative period, and their regulatory networks remain to be elucidated. Furthermore, most of these studies focused on single perioperative period to explore the surgery and anesthesia‐related pathological processes and could have different results due to different perioperative period. For example, the BBB lesion hallmark MMP2 was significantly higher at 1 day after surgery, but not at 3 days after surgery.^9^, ^10^ The biological rhythm was disturbed within 3 days after surgery but fully realigned over 1 week.^11^, ^12^ Thus, it is crucial to conduct multiperiod analysis for PND and related pathological processes.

CNS gene expression is the essence of the abovementioned pathological processes, and whole‐transcriptome sequencing (RNA‐seq analysis) provides technical supports to understand the overall gene expressions and mechanisms during CNS diseases.^13^ In the present study, overall gene expressions and involved pathological processes were studied in the different perioperative periods. The potential signaling pathways and gene modulation networks were established, which included synapse and neuronal alteration, metabolic disorder, oxidative stress, BBB damage, neurotoxicity, and cognitive dysfunction. These results provide an insight into the overall mechanisms of PND, as well as valuable therapeutic gene targets during different periods.

## 2 METHODS

### 2.1 Animals

The animal experiments were performed in accordance with the guide for the care and use of laboratory animals and the protocol was approved by the local biomedical ethics committee (No. LA2018085). Female C57BL/6 mice, 18‐month‐old, weighing between 23 and 34 g were used. The mice were housed in cages and maintained on a standard housing condition with food and water ad libitum for 2 weeks. Since POD commonly occurs within postoperative days 2–5^4^ and postoperative day 2 is a typical time point for POD or POCD study, it was selected as a study time point. As POCD normally occurred within 1 postoperative week, and the neuropsychological tests were normally carried out at postoperative day 7,^14^ it was selected as another study time point. Thus, 4 study time points were chosen: control condition (Con, preoperative time point), 30 min after surgery (Day0, postoperative day 0, the time point between intraoperative period and postoperative period), postoperative day 2 (Day2), and postoperative day 7 (Day7). The perioperative period was divided into intraoperative period (between Con and Day0), early postoperative period (between Day0 and Day2), and late postoperative period (between Day2, and Day7). Mice was randomly assigned to Con, Day0, Day2, and Day7 groups (*n* = 6).

### 2.2 Surgery and anesthesia

Minimum alveolar concentration of sevoflurane for mice has been reported as 2.4%–2.7%.^15^ In the present study, mice in Day0, Day2, and Day7 groups received 2.5% sevoflurane in 50% oxygen for 30 min through breathing masks, and the control group received 50% oxygen for 30 min. The mice breathed spontaneously, and the sevoflurane concentration was monitored continuously with an anesthetic monitor (Datex, Tewksbury, MA, USA). The surgical procedure (exploratory laparotomy) was modified from previous studies^16^ and performed for the 3 groups. A longitudinal midline incision was made from xiphoid to 0.5‐cm proximal pubic symphysis on the skin. The abdominal muscles and peritoneum, then approximately 10 cm of the intestine were exteriorized. The bowel loops remained outside the abdominal cavity for 1 min and then replaced into the abdominal cavity. The incision was finally sutured layer by layer with 5–0 Vicryl thread. The entire procedure was completed under sevoflurane anesthesia. The rectal temperature was maintained at 37 ± 0.5°C, and this surgical protocol has been shown not to significantly alter values of blood pressure and blood gas in the preliminary studies. Then the mice were put into a chamber containing 50% oxygen until 10 min after the recovery of consciousness. Mice in Day0, Day2, and Day7 groups were sacrificed by decapitation 30 min, 2 days and 7 days after surgery, respectively. The brain tissue was removed rapidly, and the hippocampus was dissected out and frozen in liquid nitrogen.

### 2.3 RNA‐Seq library preparation and sequencing analysis

Total RNAs were isolated from the hippocampus using TRIzol reagent (Invitrogen, Carlsbad, CA, USA), then digested with RNase‐Free DNase to remove residual DNAs. The Quantity and purity were detected with Nanodrop 2000 (Thermo Fisher, Wilmington, DE, USA) and Qubit Fluorometer (Invitrogen, Carlsbad, CA, USA). Library construction was performed according to the Illumina sample preparation for RNA‐seq protocol. The mRNA was enriched by magnetic beads with Oligo (dT) after the samples were qualified. When the enrichment was complete, the mRNA was interrupted into short segments with the addition of a fragmentation buffer. Subsequently, double‐stranded cDNA was synthesized by reverse transcription using 6‐base random primers. The purified double‐stranded cDNA was subjected to terminal reparation, singe nucleotide A (Adenine) addition and serial sequencing. The fragment size of double‐stranded cDNA was selected by an AMpure XP bead (Beckman coulter, Shanghai, China), and the selected double‐stranded cDNA was subjected to PCR enrichment to construct a cDNA library. Constructing and sequencing the RNA‐seq library for each sample was conducted (Compass Biotechnology, Beijing, China) based on the protocols of Illumina HiSeqTM2500/MiSeq™ to generate paired‐end reads (150 bp in length). The quality of RNA‐seq reads from all the brain tissues was checked using FastQC (v0.11.5, Babraham institute, Cambridge, UK).

### 2.4 DEGs identification and GO/KEGG analysis

The abundance of transcription was the direct indicator of gene expressions, and RPKM can compare expression level through RPKM gene distribution. Differences in gene expressions among groups were analyzed by HTSeq v0.5.4p3. Read count data were standardized with TMM, the significance (*p* < 0.05) and fold change were set, and the differences in expression were analyzed by DEGseq (v1.34.0). The overall distribution of the differential genes was shown by Volcano plot. Gene Ontology (GO) functional annotation and Kyoto Encyclopedia of Genes and Genomes (KEGG) pathway enrichment analyses were performed for DEGs using Database for Annotation, Visualization, and Integrated Discovery (DAVID, https://david.ncifcrf.gov). GO enrichment analysis contains 3 categories: biological process, molecular function and cellular component.

### 2.5 Quantitative real‐time PCR (qPCR)

The qPCR was performed on the CFX96 Real‐Time PCR Detection System (Bio‐Rad, Hercules, CA, USA). Amplification mixture consisted of PowerUpTM SYBRr Green master mix (Thermo Fisher, Wilmington, DE, USA), 10 μM forward and reverse primers (Invitrogen, Carlsbad, CA, USA) and approximately 1.5 μl of cDNA template. Primer sequences were obtained from the literature and checked for their specificity through in silico PCR. The forward and reverse primers are shown in Table 1. Amplification was carried out with an initial denaturation step at 95°C for 2 min, followed by 45 cycles of 95°C for 10 s, 55°C for 30 s, and 60°C for 30 s, then 65°C for 2 min in 10 μl reaction volume. All reactions were run in duplicate and the results were averaged from 6 independent studies. qPCR was quantified in 2 steps. First, β‐actin levels were used to normalize target gene levels (ΔCt = Ct_target gene_‐Ct_β‐actin_, target gene level = 2^‐ΔCt^). Second, the target gene levels of the sevoflurane group were presented as the percentage of those of the control group, and 100% of the target gene levels referred to the control levels.

**TABLE 1:** The forward and reverse primers for qPCR

Genes	Primers	Sequence (5′–3′)	Genes	Primers	Sequence (5′–3′)
*Klf4*	Forward primer	AGCAGGTGCCCCGACTAA	*Cry2*	Forward primer	TGACCTAGACAGAATCATCGAACT
	Reverse primer	TCCTGGTGGGTTAGCGAGTT		Reverse primer	CAAGTCCTTCCGTGGGGAAT
*Ezh2*	Forward primer	CAACCCGAAAGGGCAACAAA	*Sirt1*	Forward primer	TGACGCTGTGGCAGATTGTT
	Reverse primer	TTTCTCGTTCGATGCCCACA		Reverse primer	CCGCAAGGCGAGCATAGATA
*Tsc2*	Forward primer	CAACTGCTTACCAGCCGAGA	*Mylip*	Forward primer	CAGGAGCAGACAAGGCATATC
	Reverse primer	CAGTGGGGCATCTTCCATGT		Reverse primer	GCTCCTTATGCTTCGCAACG
*Mtor*	Forward primer	CTCTCTGACCCAGTTCGTCC	*Gpld1*	Forward primer	AAGTGTGAGGTGAGGATATTGGAG
	Reverse primer	GCCAAGACACAGTAGCGGA		Reverse primer	TCGGTGTGTTCCCTCTACAC
*Map1a*	Forward primer	TGATCAGGACTTCTTCCGCC	*Ptpn23*	Forward primer	CATGATCTGGCTGGACCTGAA
	Reverse primer	AGGACCAGGACGTTCAGTTG		Reverse primer	GGCACCCGACTCTGTAGGTA
*Rims1*	Forward primer	GTATTGGCGTAGTGCCTCCA	*Mmp2*	Forward primer	CCCCATGAAGCCTTGTTTACC
	Reverse primer	AGCGGTGATGTGTGGTTCTT		Reverse primer	AAGACACATGGGGCACCTTC
*Arf6*	Forward primer	CAATGACCGGGAGATGAGGG	*Plec*	Forward primer	CTGGAAGGTGCTCAGTGGTT
	Reverse primer	GAGGGCTGCACATACCAGTT		Reverse primer	AACGTGACTAGGGACCAGGA
*Dvl3*	Forward primer	GCGGCCCAGCTATAAGTTCT	*Cldn5*	Forward primer	CAGTTAAGGCACGGGTAGCA
	Reverse primer	GATACCAGCCAGGACACCAC		Reverse primer	GGCACCGTCGGATCATAGAA
*Celsr1*	Forward primer	AATGACGCCCTCAAGGTCAG	*Lrp1*	Forward primer	GGCGGTGTGACAACGACAAT
	Reverse primer	TTCAGGAGACACGCATCCAC		Reverse primer	GGCACTGGAACTCATCCGAG
*Htr1a*	Forward primer	TACTCCACTTTCGGCGCTTT	*Trim8*	Forward primer	AAGATCGGCCACCTGAACTC
	Reverse primer	GGCTGACCATTCAGGCTCTT		Reverse primer	TACGCTCTGTAGGAAGGGCA
*Htr1b*	Forward primer	TCCCTGCCCCGTTTTGTATC	*Ncam1*	Forward primer	GCCAGACAGAGCATCGTGAA
	Reverse primer	ACAGAGTTCTCCCCAGAGCA		Reverse primer	CAGACGTATTCGGCCTCGTC
*Ppara*	Forward primer	CTGGGCAAGAGAATCCACGA	*Nf1*	Forward primer	AGCTGTAGGCCAAACCAGTC
	Reverse primer	CGTCTTCTCGGCCATACACA		Reverse primer	CATAGTCAGTCTCTGCCACTCT
*Micu1*	Forward primer	TCGCGCTCTTTGACTGTGAT	*Slc8a3*	Forward primer	CCATCGGGCTCAAGGATTCG
	Reverse primer	TTTCCACATGGCCTGCATGA		Reverse primer	TTACTGCCTGTGACGTTGCC
*Itpr1*	Forward primer	GAGCTTGAACCAAGTCCACCC	*Srf*	Forward primer	CGGCGCTACACGACCTT
	Reverse primer	CTCACCCCTGCTTGTGGAAC		Reverse primer	TGGCACTCATTCTCTGGTCTG
*Fbxl3*	Forward primer	TGTCGCAGCTTGTGAATTGC	*Gapdh*	Forward primer	ACTCTTCCACCTTCGATGCC
	Reverse primer	GCTTGAGTGTGTCGCTGTTG		Reverse primer	TGGGATAGGGCCTCTCTTGC
*Fbxl21*	Forward primer	GCGTTCGTCACGCAGAGTT			
	Reverse primer	GGGGTAATCACCGACACCCA			

### 2.6 Immunofluorescence analysis

Immunofluorescence was performed to determine the gene expressions, as described in our previous studies.^17^ The hippocampus was fixed with 4% paraformaldehyde for 24 h, cryoprotected with 30% sucrose for 48 h, and sectioned using a cryostat (Cryotome E, Thermo Fisher, Waltham, MA, USA). Coronal sections (10 μm thickness) were incubated with ARF6 antibody (1:100 dilution; Abcam, Cambridge, UK), SIRT1 antibody (1:50 dilution; Abcam, Cambridge, UK), or MMP2 antibody (1:200 dilution; Abcam, Cambridge, UK) overnight at 4°C, followed by incubation with a goat anti‐rabbit conjugated CY3 antibody (1:300 dilution; Servicebio, Wuhan, China) for 50 min at room temperature. Nuclei were subsequently counterstained with DAPI (Servicebio, Wuhan, China) for 10 min at room temperature. Images were captured using a Nikon Eclipse Ti confocal microscope. Hippocampal subregions CA1 and DG were analyzed for ARF6, SIRT1, and MMP2 expressions.

### 2.7 Fear conditioning test (FCT) and Morris water maze

The FCT (Xeye CPP, MacroAmbition S&T Development, Beijing, China) was used to assess the cognitive function of mice after surgery as described in previous studies.^17^ FCT consisted of a training process at 3 h after surgery and evaluations at 2 and 7 days after surgery. In the training process, mice were placed in the context chamber to acclimate for 180 s, then they received a 2 Hz pulsating tone (80 dB, 3600 Hz) for 60 s co‐terminated with a mild foot shock (0.8 mA, a 0.5 s). In the evaluations, the hippocampus‐dependent memory was assessed by the freezing time during exposure to a novel context test (the test was performed in the same chamber but with no cues or shock), while the hippocampus‐independent memory was assessed by the freezing time during exposure to the tone stimulus (the test was performed in an alternative context and with no shock).

The Morris water maze test (Sunny Instruments Co. Ltd., Beijing, China) was used to assess the spatial learning and memory of mice after surgery. Morris water maze test consisted of a circular tank (120 cm in diameter and 50 cm high) containing water (23 ± 1°C) that is divided into four quadrants and a platform (10 cm in diameter) located 1 cm below the water in the target quadrant. In the place navigation test, the mice were placed in one quadrant facing the wall of the maze and allowed to explore for the hidden platform for 90 s in each trial (four trials per day with an intertrial interval of 5 min). The time to locate the submerged plat form was recorded (defined as the escape latency). If the platform was not found within 90 s, the mice were guided to the platform, where they stayed for 15 s. Mice underwent daily testing in the water maze from day 1 to day 5 after surgery. On postoperative day 6, the submerged platform was removed from the water maze and a spatial probe test was performed for 90 s. The swimming speed, escape latency, times of platform crossing, and the time spent in target quadrant were recorded by a video camera.

### 2.8 Statistical analysis

Statistical analysis was performed with GraphPad Prism 7.0 software. Quantitative data are presented as the mean ± SD. D'Agostino & Pearson omnibus normality test were used to assess normality of data, and test results exhibited a normal/Gaussian distribution. Non‐paired two‐tailed Student's *t*‐test was used to identify significant differences between the 2 groups. One‐way ANOVA with Bonferroni's multiple comparison test was utilized to analyze significant differences between multiple groups. *p* < 0.05 was considered significant. The *p*‐value was adjusted with the FDR method (Benjamini Hochberg procedure). The significance of GO and KEGG enrichment was calculated by the hypergeometric distribution and Fisher exact test, and the specific term was more significantly enriched with a lower *p*‐value.

## 3 RESULTS

### 3.1 Gene expression patterns in 3 perioperative periods

**FIGURE 1:**
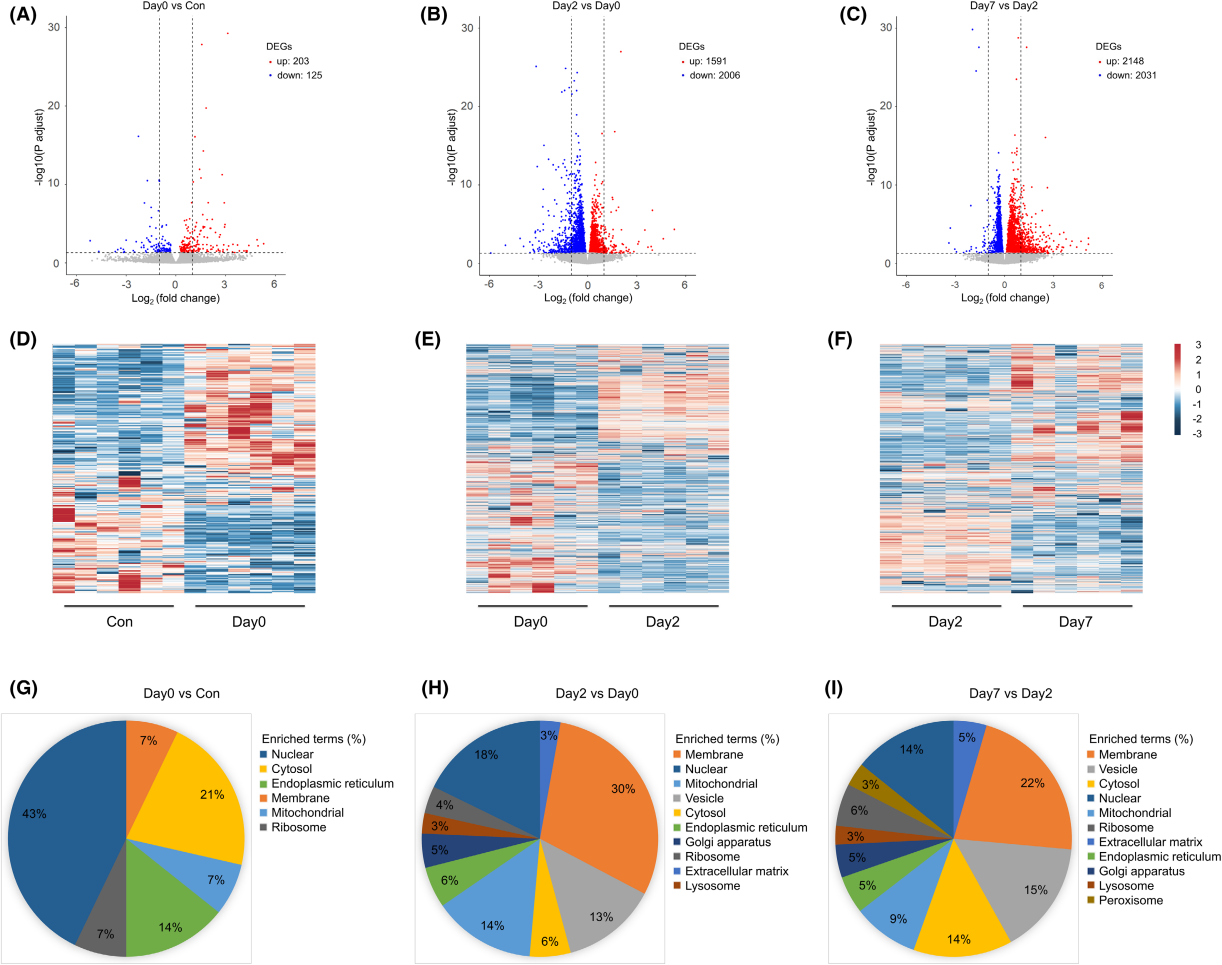
(A–C) The differentially expressed genes (DEGs, *p* < 0.05) in intraoperative period (Day0 group vs. Control group), early postoperative period (Day2 group vs. Day0 group) and late postoperative period (Day7 group vs. Day2 group). Red dots are upregulated genes and blue ones are downregulated genes. (D–F) Heatmaps show the expression patterns of DEGs in 3 periods. Red rectangles are upregulated genes and blue ones are downregulated genes. The deep color means significant difference. (G–I) Pie charts show the percentages of DEG cellular locations in 3 periods

The hippocampi of aged mice were obtained from Con, Day0, Day2, and Day7 groups (*n* = 6). Then DEGs in intraoperative period (Day0 vs. Con), early postoperative period (Day2 vs. Day0), and late postoperative period (Day7 vs. Day2) were collected and analyzed. The volcano plot and heatmap were firstly generated. In Figure 1A–C, highlighted red dots were genes with significantly upregulated expression (*p* < 0.05), highlighted blue dots were genes with significant downregulated expression (*p* < 0.05). In intraoperative period, there were 328 DEGs (*p* < 0.05), 125 of which were downregulated and 203 were upregulated (Figure 1A). In early postoperative period, there were 3597 DEGs (*p* < 0.05), 2006 of which were downregulated and 1591 were upregulated (Figure 1B). In late postoperative period, there were 4179 DEGs (*p* < 0.05), 2031 of which were downregulated, and 2148 were upregulated (Figure 1C). There are more DEGs in postoperative periods, which may attribute to the length of the periods.

Heatmap showed the expression patterns of DEGs (*p* < 0.05) in 3 periods, and each column indicated 1 hippocampus sample. Red rectangles were upregulated genes and blue ones were downregulated genes, and deep color referred to significant difference (Figure 1D–F). The means of log2 fold change of top 10% and 20% DEGs of intraoperative period were higher than those of postoperative periods, which indicated that the differences of DEGs of intraoperative period were more significant than postoperative periods. Based on GO database, the percentages of DEG cellular locations in 3 periods were displayed with pie charts. In intraoperative period, the DEGs mainly located at Nuclear, Cytosol, Endoplasmic reticulum (43%, 21%, and 14%, respectively, Figure 1G). In early postoperative period, the DEGs mainly located at Membrane, Nuclear, Mitochondrial (30%, 18%, and 14%, respectively, Figure 1H). In late postoperative period, the DEGs mainly located at Membrane, Vesicle, Cytosol (22%, 15%, and 14%, respectively, Figure 1I). These results indicated that nuclear, cytosol and endoplasmic reticulum were involved firstly. After surgery, more cellular organelles and structures were involved, which included membrane, mitochondria, vesicle, and cytosol.

### 3.2 DEG analysis revealed 9 main neuropathological processes in 3 perioperative periods

**FIGURE 2:**
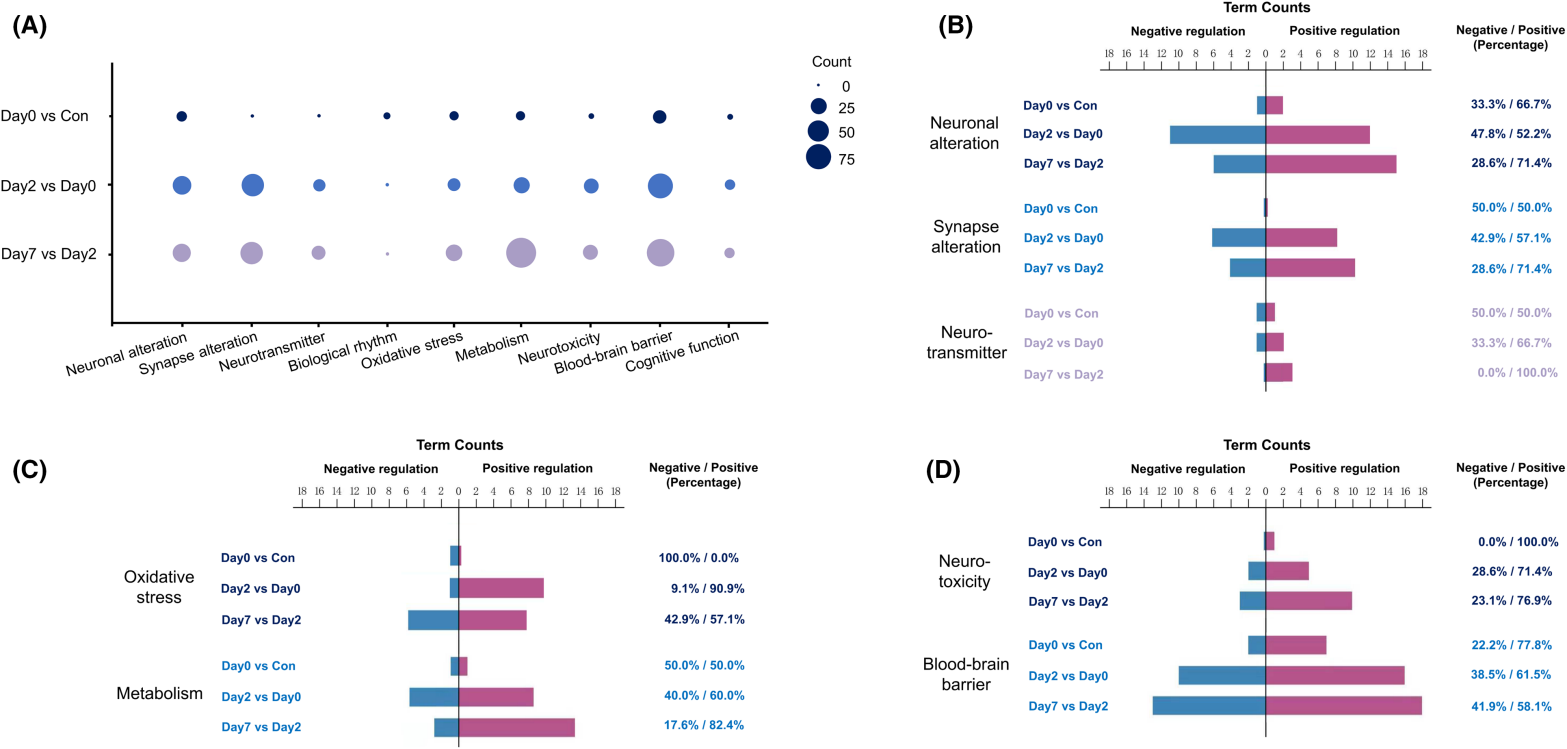
Gene Ontology (GO) enrichment analysis of DEGs. (A) The number of BP terms of 9 categories in 3 periods are shown as the circle area. (B–D) The counts and percentages of negative regulation terms (blue) and positive regulation terms (purple) of all categories in 3 periods

Biological process (BP) terms were identified based on GO database. Considering the perioperative pathological features, processes with top count were divided into 9 categories. They were Neuronal alteration, Synapse alteration, Neurotransmitter, Biological rhythm, Oxidative stress, Metabolism, Neurotoxicity, Blood–brain barrier, and Cognitive function. The number of BP terms in 3 periods of 9 categories was shown as the circle area. In intraoperative period, the categories with top term numbers were Blood–brain barrier, neuronal alteration, oxidative stress, and metabolism. In postoperative periods, the categories with top term numbers were metabolism, blood–brain barrier, synapse alteration, neuronal alteration, oxidative stress, and neurotoxicity. For most categories, there were more BP terms in postoperative periods, however, for Biological rhythm, there were more BP terms in intraoperative period (Figure 2A).

Figure 2B–D showed negative regulation terms (blue) and positive regulation terms (purple) in abovementioned categories. Negative and positive regulation terms were categorized based on their effects. For example, negative terms of Blood–brain barrier in late postoperative period contained negative regulation of endothelial cell proliferation, negative regulation of epithelial to mesenchymal transition, negative regulation of angiogenesis, negative regulation of sprouting angiogenesis, negative regulation of cell junction assembly, epithelial cell apoptotic process, etc. Positive terms contained prostate gland epithelium morphogenesis, positive regulation of blood vessel endothelial cell migration, vascular endothelial cell proliferation, etc. The percentages of negative and positive terms were 41.9% and 58.1%, respectively. In intraoperative period, positive regulations were the main directions for Neuronal alteration, Neurotoxicity, and Blood–brain barrier, and negative regulations were the main direction for Oxidative stress. In postoperative periods, positive regulations were the main directions for most categories including oxidative stress; however, negative regulations also existed, such as negative regulation of cell development and neurogenesis, negative regulation of neurotransmitter transport. The results indicated the roles of these categories during perioperative pathological processes in the hippocampus. There were no obvious negative or positive regulation terms in Biological rhythm and Cognitive function.

**FIGURE 3:**
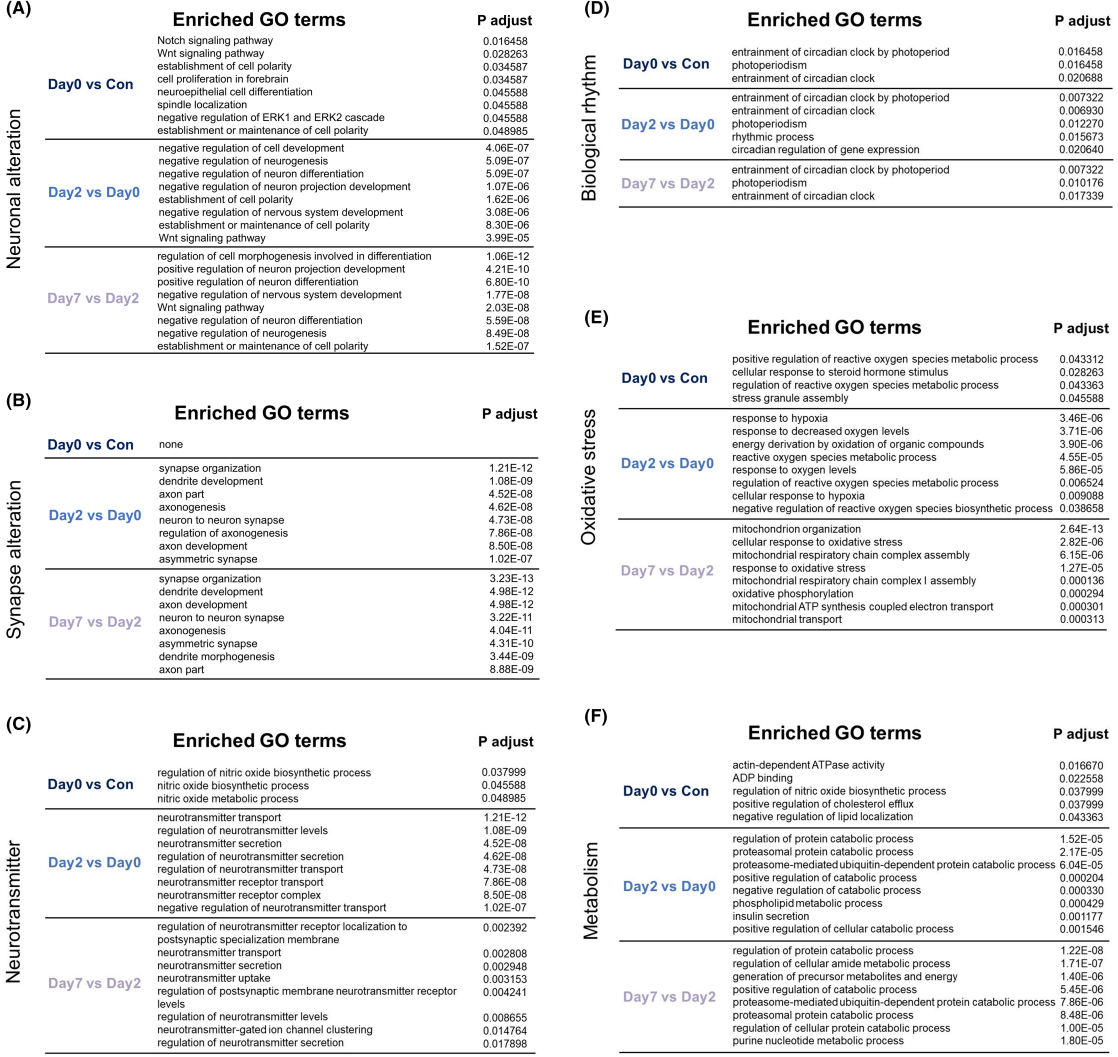
The lists of top enriched GO terms of 6 categories in 3 periods (ranked by P adjust)

**FIGURE 4:**
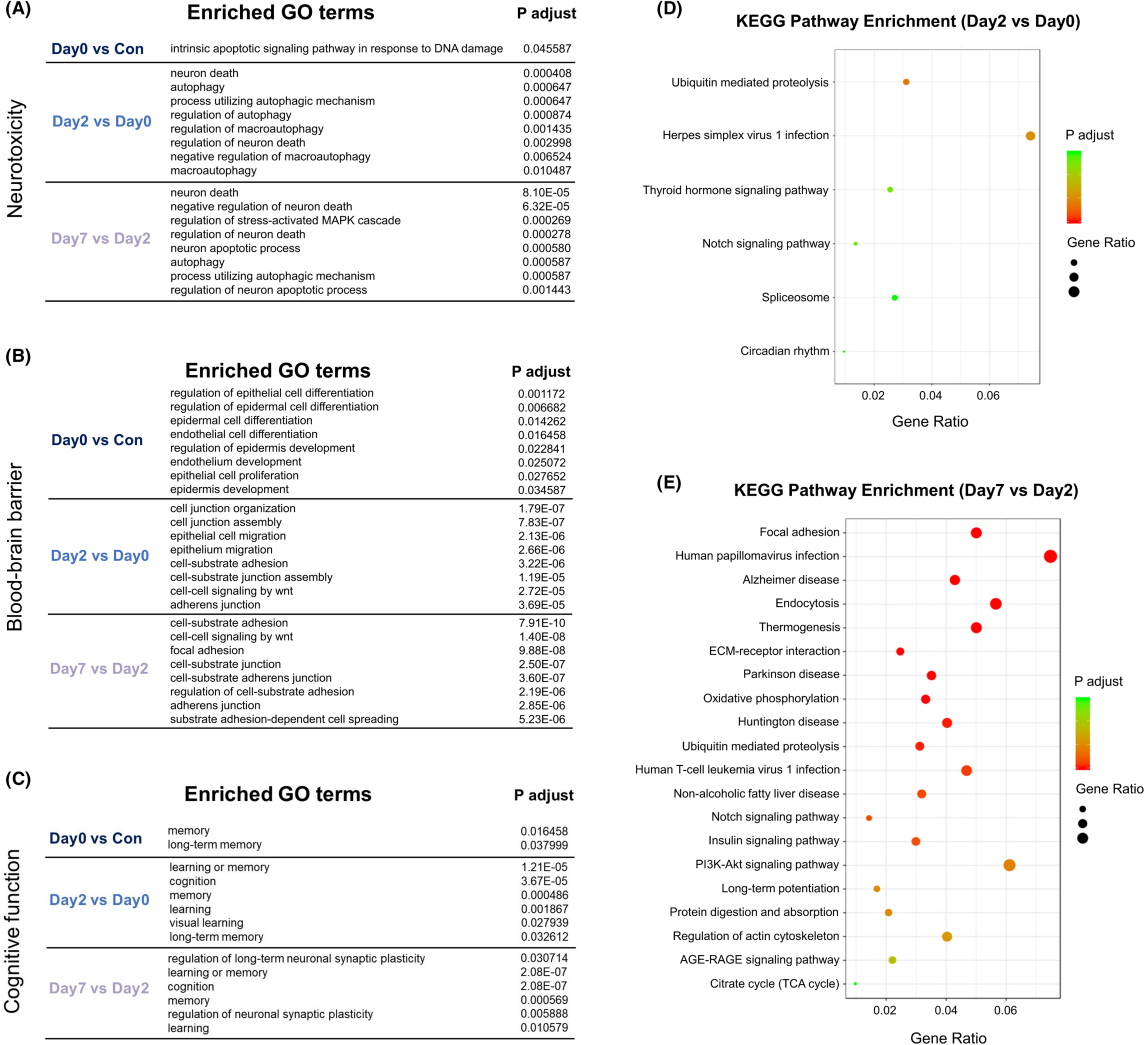
(A–C) The lists of top enriched GO terms of 3 categories in 3 periods (ranked by P adjust). (D–E) Bubble charts show KEGG pathway enrichment analysis of DEGs in early postoperative period (Day2 vs. Day0) and late postoperative period (Day7 vs. Day2). Color in red means low P adjust and the area of bubbles means the gene count

To be specific, the top 8 BP terms in each category were listed in Figures 3A–F and 4A–C, ranked by *p*‐value. The enriched terms of Neuronal alteration, Synapse alteration, and Neurotransmitter were mainly in postoperative periods. For Neuronal alteration, the top terms in early postoperative period were negative regulation of cell development and neurogenesis, however, in late postoperative period were positive regulation of neuron projection development and differentiation. For Synapse alteration, the top terms in postoperative periods were synapse organization and dendrite development. For Neurotransmitter, the top terms in early postoperative period were neurotransmitter transport and regulation of neurotransmitter levels, and in late postoperative period were regulation of neurotransmitter receptor localization to postsynaptic specialization membrane and neurotransmitter transport (Figure 3A–C).

For Biological rhythm, the enriched terms were mainly in intraoperative period, and the top terms were entrainment of circadian clock by photoperiod and photoperiodism. For Oxidative stress, the top terms in intraoperative period were positive regulation of reactive oxygen species metabolic process and cellular response to steroid hormone stimulus, in early postoperative period were response to hypoxia and decreased oxygen levels, and in late postoperative period were mitochondrion organization and cellular response to oxidative stress. For Metabolism, the top terms in intraoperative period were positive regulation of cholesterol efflux and negative regulation of lipid localization, in early postoperative period included both positive and negative regulation of catabolic process, and in late postoperative period included only positive regulation of catabolic process (Figure 3D–F).

For Neurotoxicity, the top terms in intraoperative period were intrinsic apoptotic signaling pathway in response to DNA damage, in early postoperative period were neuron death and autophagy, and in late postoperative period were neuron death and negative regulation of neuron death. For Blood–brain barrier, the top terms in intraoperative period were regulation of epithelial and epidermal cell differentiation, in early postoperative period were cell junction organization and assembly, and in late postoperative period were cell‐substrate adhesion and cell–cell signaling by Wnt. For Cognitive function, the top terms in 3 periods included memory, long‐term memory, regulation of long‐term neuronal synaptic plasticity, etc. (Figure 4A–C).

KEGG pathway analysis revealed the enriched signaling pathways. Top enriched signaling pathways in early postoperative period included ubiquitin mediated proteolysis, herpes simplex virus 1 infection, thyroid hormone signaling pathway, etc. (Figure 4D). Top enriched signaling pathways in late postoperative period included focal adhesion, human papillomavirus infection, Alzheimer disease, etc. (Figure 4E). However, there were no significant enriched signaling pathways in intraoperative period, which may attribute to the short time interval and limited DEGs in the period.

### 3.3 DEG intersections and TF regulations in 9 perioperative neuropathological processes

**FIGURE 5:**
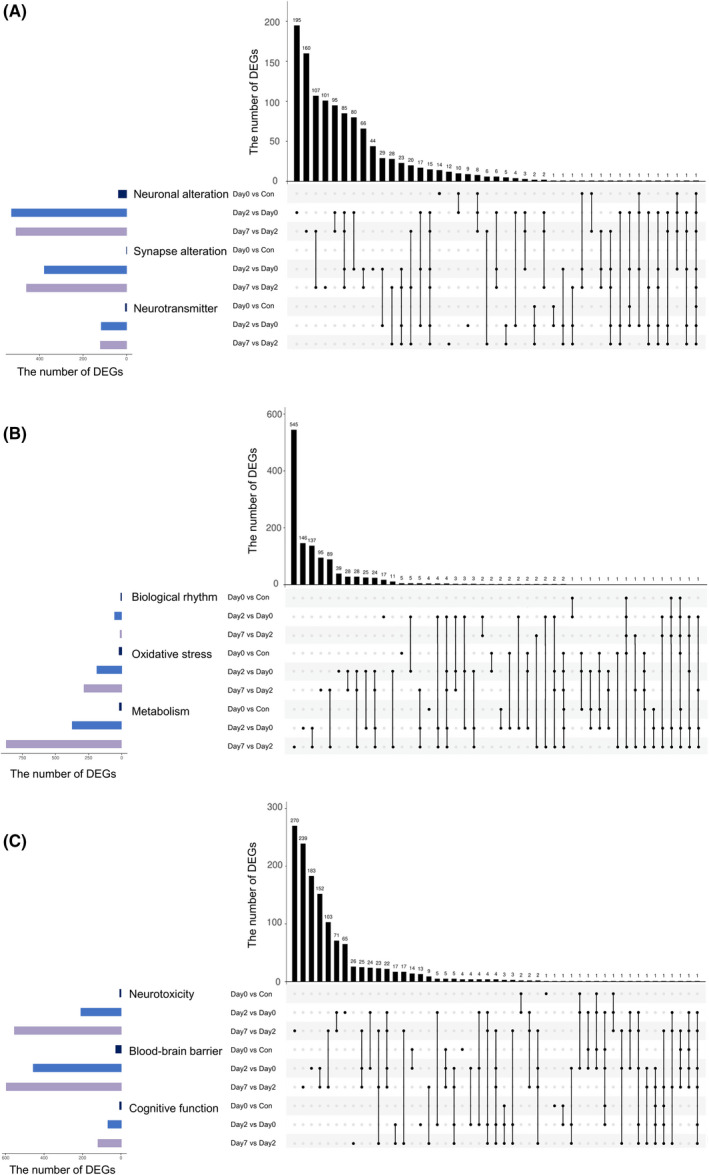
UpSet plots show the intersections of DEGs among neuronal alteration, synapse alteration and neurotransmitter (A), among biological rhythm, oxidative stress and metabolism (B), and among neurotoxicity, blood–brain barrier and cognitive function (C). The left bar chart indicates total number of DEGs for each category, the bottom dark connected dots indicate substrates for each intersection, and the top bar chart indicates intersection sizes between DEGs

Figure 5A showed the intersections of DEGs among Neuronal alteration, Synapse alteration and Neurotransmitter. The categories with top DEG numbers were Neuronal alteration in early and late postoperative periods, and Synapse alteration in late postoperative period (*n* = 195, 160, and 101, respectively). The DEG intersections with largest size were between Neuronal alteration and Synapse alteration, and between Neurotransmitter and Synapse alteration in postoperative periods. Figure 5B showed the intersections of DEGs among Biological rhythm, Oxidative stress and Metabolism. The categories with top DEG numbers were Metabolism and Oxidative stress in late and early postoperative periods (*n* = 545, 146, 95, and 39, respectively), which accounted for 93.8% of all DEGs. The DEG intersections with largest size were between Oxidative stress and Metabolism in postoperative periods. The DEG intersections with other biological processes were relatively rare. Figure 5C showed the intersections of DEGs among Neurotoxicity, Blood–brain barrier and Cognitive function. The categories with top DEG numbers were Neurotoxicity, Blood–brain barrier and Cognitive function in late postoperative period, as well as Neurotoxicity and Blood–brain barrier in early postoperative period (*n* = 270, 239, 95, 163 and 65, respectively). The DEG intersections with largest size were between Neurotoxicity and Blood–brain barrier in postoperative periods. For Cognitive function, the DEG intersections were relatively rare and mainly in late postoperative period.

To better manifest the role of DEGs in perioperative period, as well as the relationship among different categories, we used 3 tables to show the top DEGs involved most periods of different categories (Tables 2, 3, 4). The numbers below categories refer to the involved periods of each DEGs, and the numbers below ‘Count’ refer to the total number of involved periods. As shown in Table 2, *Klf4* was differentially expressed in 8 periods of Neuronal alteration, Synapse alteration and Neurotransmitter. It could regulate ApoE, promote inflammation through NF‐κB pathway, and trigger the cellular response to DNA damage.^18^ Fifteen genes were differentially expressed in 6 periods, including *Shank1*, *Rims1*, *Shank2*, etc., which involved in multiple neuron related processes. Four genes were differentially expressed in 4 or 5 periods. As shown in Table 3, *Cry2* was differentially expressed in 7 periods of Biological rhythm, Oxidative stress and Metabolism, and it has been reported to be involved in circadian rhythm and metabolism regulations.^19^ *Klf4* and *Khsrp* were differentially expressed in 6 periods. Eight genes were differentially expressed in 5 periods, including *Per1*, *Sik1*, *Icam1*, etc. Nine genes were differentially expressed in 4 periods. As shown in Table 4, *Ptprz1* was differentially expressed in 6 postoperative periods of Neurotoxicity, Blood–brain barrier and Cognitive function, and it could negatively regulate oligodendrocyte precursor proliferation.^20^ Eight genes were differentially expressed in 5 periods including *Itpr3*, *Slc7a11*, *Icam1*, etc., and they were involved in multiple processes of neurotoxicity and cognitive dysfunction.^21^ Nine genes were differentially expressed in 4 periods.

**TABLE 2:** Top 20 DEGs involved in neuronal alteration, synapse alteration and neurotransmitter related function terms at different periods

DEGs	Count	Neuronal alteration	Synapse alteration	Neuro‐transmitter	DEGs	Count	Neuronal alteration	Synapse alteration	Neuro‐transmitter
*Klf4*	8	1,2,3	2,3	1,2,3	*Scrib*	6	2,3	2,3	2,3
*Shank1*	6	2,3	2,3	2,3	*Rab5a*	6	2,3	2,3	2,3
*Rims1*	6	2,3	2,3	2,3	*Vamp7*	6	2,3	2,3	2,3
*Shank2*	6	2,3	2,3	2,3	*Slc7a11*	6	2,3	2,3	2,3
*Rap1b*	6	2,3	2,3	2,3	*Hsp90aa1*	6	2,3	2,3	2,3
*Synj2p*	6	2,3	2,3	2,3	*Ppp3r1*	6	2,3	2,3	2,3
*Celsr1*	6	2,3	2,3	2,3	*Chrna3*	5	3	2,3	2,3
*Dlg4*	6	2,3	2,3	2,3	*Grin2c*	5	2	2,3	2,3
*Dag1*	6	2,3	2,3	2,3	*Kcnj10*	4	2,3	/	2,3
*Brsk1*	6	2,3	2,3	2,3	*Slc1a3*	4	2,3	/	2,3

*Notes*: Count: the number of terms that the DEGs are involved in, 1 means intraoperative period, 2 means early postoperative period, 3 means late postoperative period.

**TABLE 3:** Top 20 DEGs involved in biological rhythm, oxidative stress and metabolism related function terms at different periods

DEGs	Count	Biological rhythm	Oxidative stress	Meta‐bolism	DEGs	Count	Biological rhythm	Oxidative stress	Meta‐bolism
*Cry2*	7	1,2,3	1	1,2,3	*Ppara*	5	2	2,3	2,3
*Klf4*	6	/	1,2,3	1,2,3	*Srebf1*	4	2	3	2,3
*Khsrp*	6	/	1,2,3	1,2,3	*Ep300*	4	2	2,3	3
*Per1*	5	1,2,3	1	3	*Hdac2*	4	2	2,3	3
*Sik1*	5	1,2,3	/	2,3	*Fbxl3*	4	2,3	/	2,3
*Icam1*	5	/	1,2,3	1,3	*Mtor*	4	/	1,2	1,2
*Per2*	5	2,3	2	2,3	*Dynll1*	4	/	2,3	2,3
*Foxo3*	5	2	2,3	2,3	*Usp19*	4	/	2,3	2,3
*Ptprn*	5	2,3	2,3	3	*Cyb5r4*	4	/	2,3	2,3
*Egfr*	5	2,3	2,3	2,3	*Rela*	4	/	2,3	2,3

*Notes*: Count: the number of terms that the DEGs are involved in, 1 means intraoperative period, 2 means early postoperative period, 3 means late postoperative period.

**TABLE 4:** Top 20 DEGs involved in neurotoxicity, blood–brain barrier and cognitive function related function terms at different periods

DEGs	Count	Neuro‐toxicity	Blood–brain barrier	Cognitive function	DEGs	Count	Neuro‐toxicity	Blood–brain barrier	Cognitive function
*Ptprz1*	6	2,3	2,3	2,3	*Ep300*	4	2,3	4	3
*Itpr3*	5	3	3	1,2,3	*Rap1b*	4	3	2,3	3
*Slc7a11*	5	2,3	2	2,3	*Rab5a*	4	3	2,3	3
*Icam1*	5	2,3	1,2,3	/	*Klf4*	4	3	1,2,3	/
*Chd8*	5	/	2,3	1,2,3	*Epha2*	4	1,2	1,2	/
*Mtor*	5	2	1,2	1,2	*Syngap1*	4	2,3	/	2,3
*Igf2*	5	3	2,3	2,3	*Ntf3*	4	2,3	/	2,3
*Egfr*	5	3	2,3	2,3	*Creb1*	4	2,3	/	2,3
*Rag1*	5	3	2,3	2,3	*Bhlhb9*	4	2,3	/	2,3
*Srf*	5	3	2,3	2,3	*Ncam1*	4	/	2,3	2,3

*Notes*: Count: the number of terms that the DEGs are involved in, 1 means intraoperative period, 2 means early postoperative period, 3 means late postoperative period.

**FIGURE 6:**
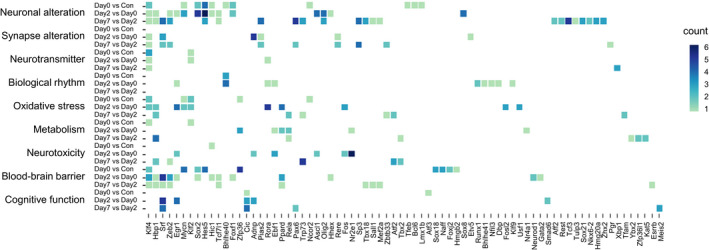
Differentially expressed transcription factors (TFs) and the counts of their related GO terms of all categories in 3 periods (E value < 0.00001). The TFs are ranked by their period participation counts of all categories (left to right)

Transcription factors (TFs) could regulate gene expressions and play roles in neurodegenerative diseases.^22^ We analyzed TF expressions, and 74 differentially expressed TFs were found during perioperative period (E value < 0.00001, Figure 6). The TFs were ranked by their period participation counts of all categories. The top 10 TFs were *Klf4*, *Hbp1*, *Srf*, *Zeb2*, *Egr1*, *Mycn*, *Klf2*, *Sox2*, *Hes5* and *Hic1*. *Klf4*‐related terms were involved in 3 periods of Neuronal alteration, Neurotransmitter and Blood–brain barrier, in 2 periods of Synapse alteration and Oxidative stress, as well as in 1 period of Metabolism. *Hbp1*‐related terms were involved in 2 periods of Neuronal alteration, Oxidative stress and Blood–brain barrier, and in 1 period of Metabolism and Neurotoxicity. *Srf*‐related terms were involved in 2 periods of Synapse alteration, Blood–brain barrier and Cognitive function, and in 1 period of Neuronal alteration. Overall, the GO terms of these TFs were most distributed in Neuronal alteration, Blood–brain barrier and Oxidative stress. Harmonizome database indicated that *Hbp1*, *Zeb2* and *Egr1* could target *Klf4*. *Klf4* could regulate target genes through phosphorylation, acetylation, methylation and ubiquitination, and show a context‐dependent function.

### 3.4 Potential modulation network construction of PND related neuropathological processes

**FIGURE 7:**
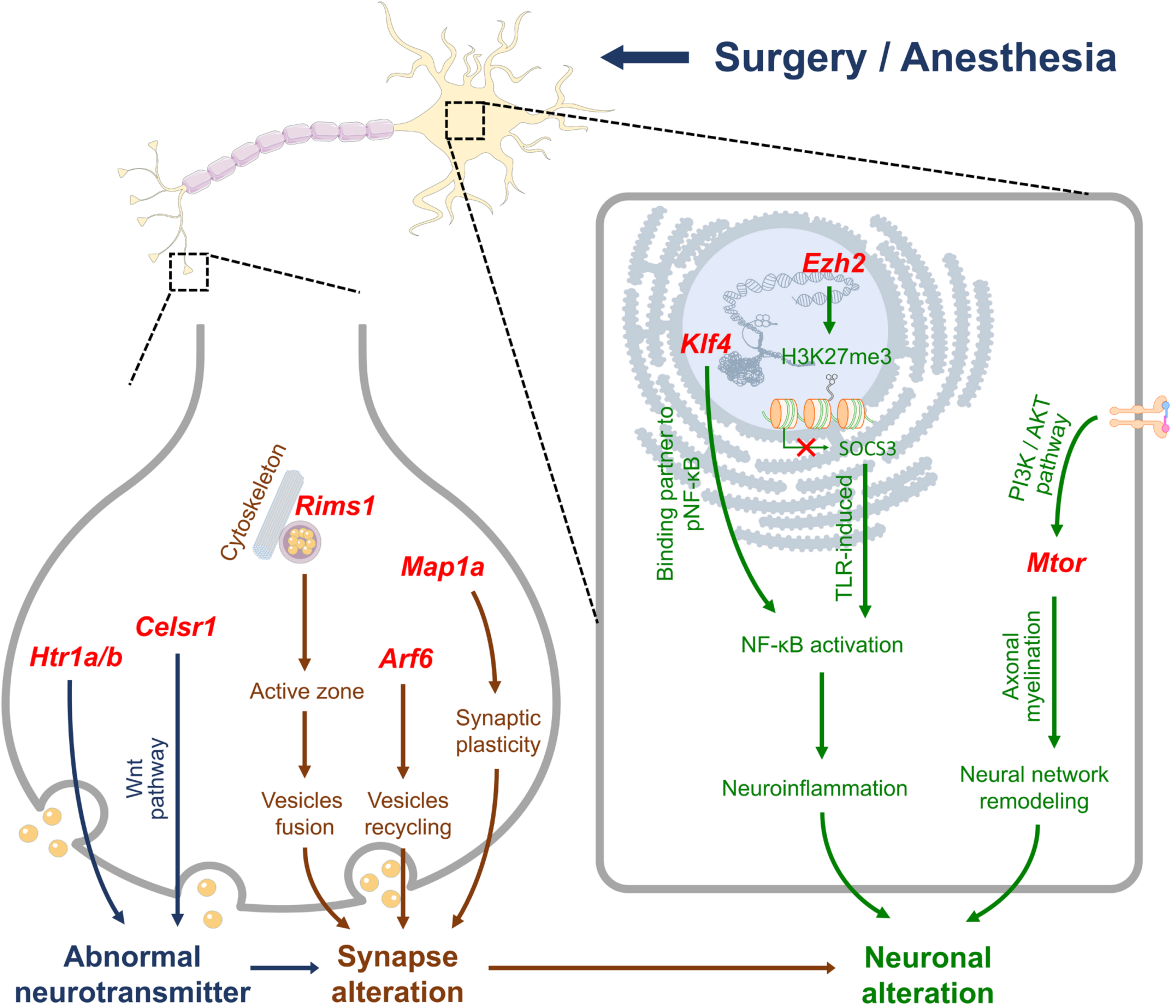
Genes (red) and hypothetical mechanisms/signaling pathways related to Neurotransmitter (blue), Synapse alteration (brown) and Neuronal alteration (green) during perioperative period

**FIGURE 8:**
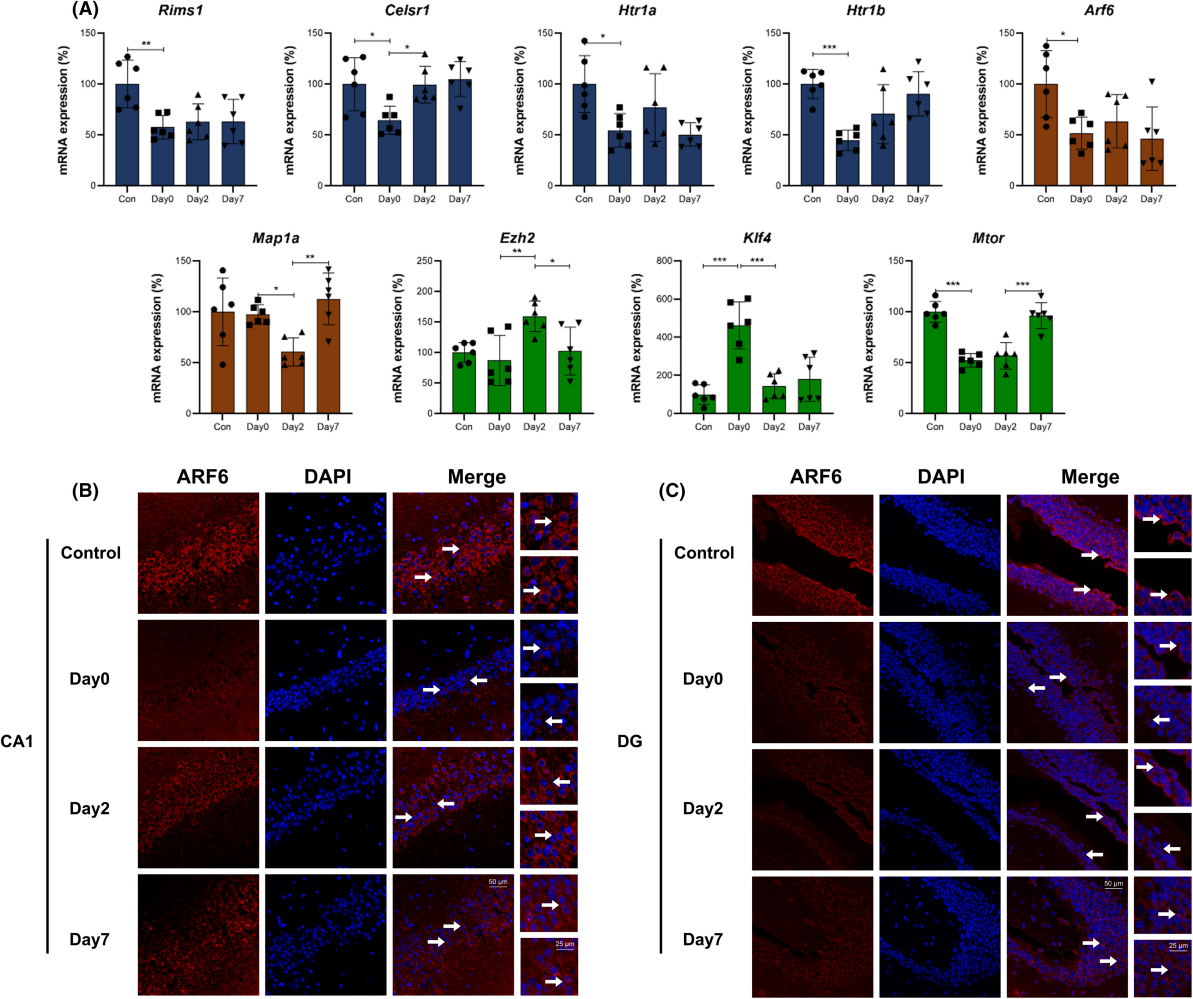
(A) qPCR verification for genes in Figure 7. The columns show gene expressions of Con, Day0, Day2, and Day7 groups (Means ± SD, *p < 0.05, **p < 0.01 and ***p < 0.001). (B and C) Immunofluorescence images show ARF6 (CY3, red) in CA1 region and DG of hippocampus. DAPI shows the nucleus in blue. Arrows point to the typical ARF6 distribution, which are provided as high magnification images on the right. Magnification 200× and 400×, scale bar 100 and 25 μm

**FIGURE 9:**
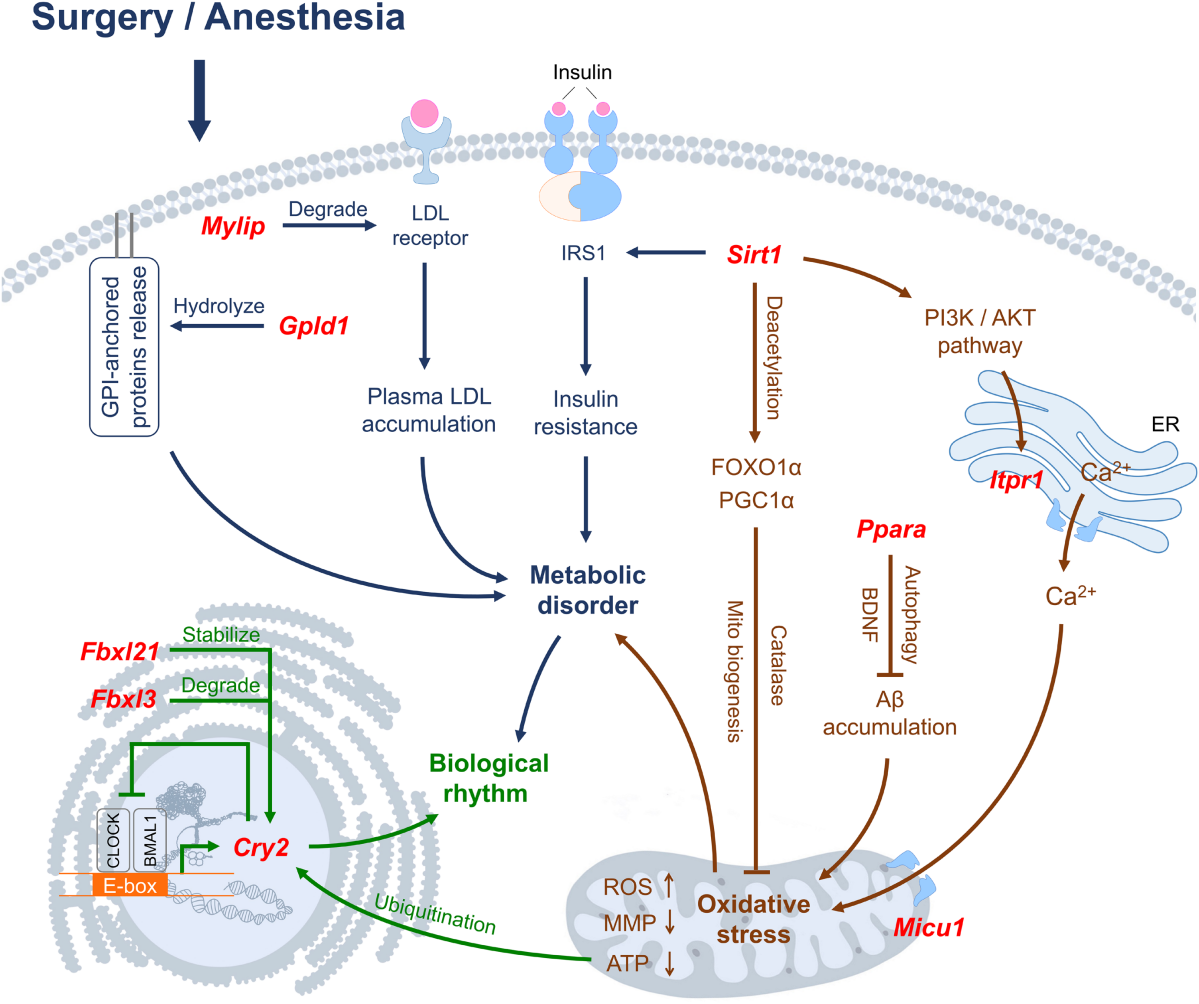
Genes (red) and hypothetical mechanisms/signaling pathways related to metabolism (blue), oxidative stress (brown) and biological rhythm (green) during perioperative period

**FIGURE 10:**
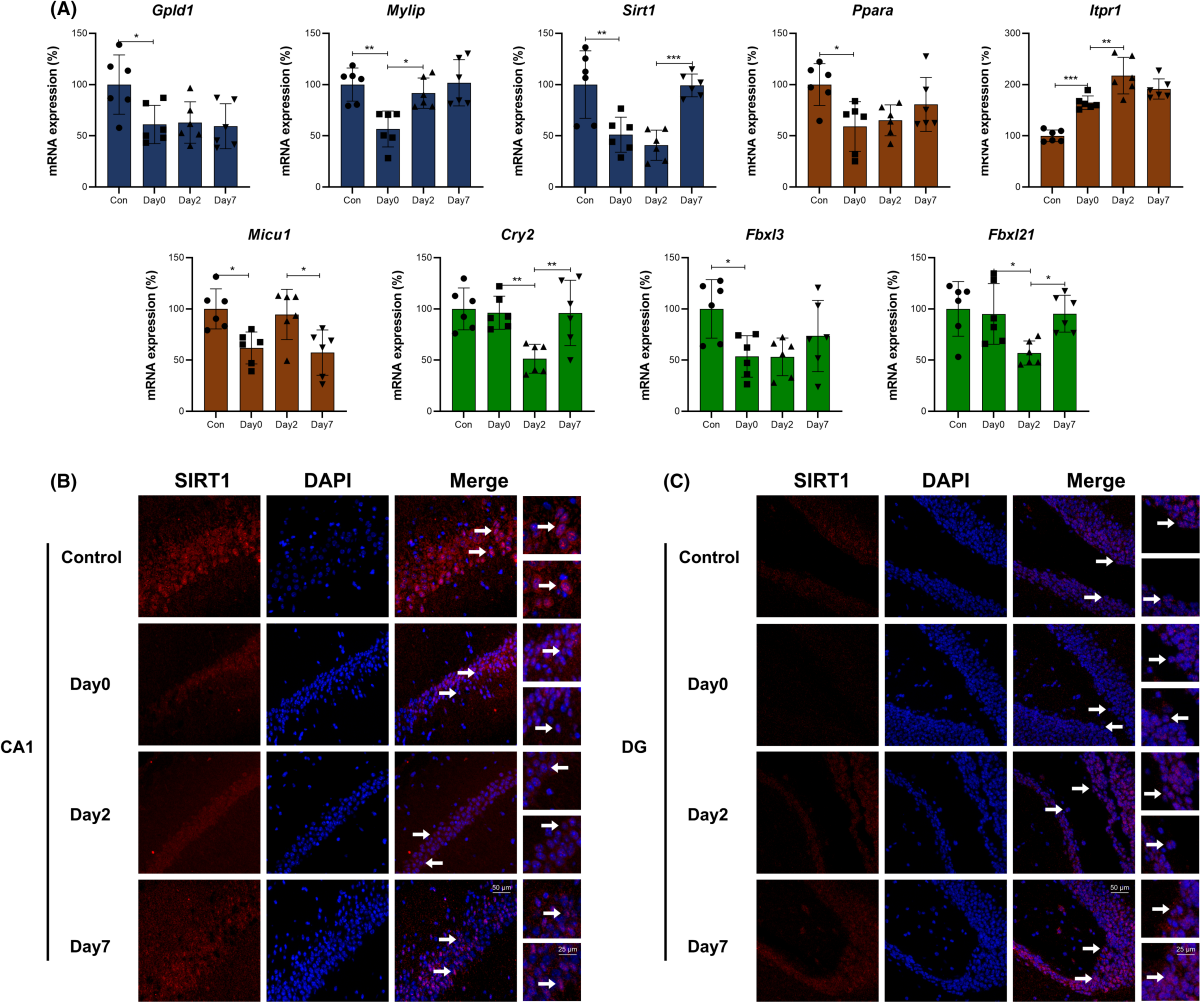
(A) qPCR verification for genes in Figure 9. The columns show gene expressions of Con, Day0, Day2, and Day7 groups (Means ± SD, *p < 0.05, **p < 0.01 and ***p < 0.001). (B and C) Immunofluorescence images show SIRT1 (CY3, red) in CA1 region and DG of hippocampus. DAPI shows the nucleus in blue. Arrows point to the typical SIRT1 distribution, which are provided as high magnification images on the right. Magnification 200× and 400×, scale bar 100 and 25 μm

**FIGURE 11:**
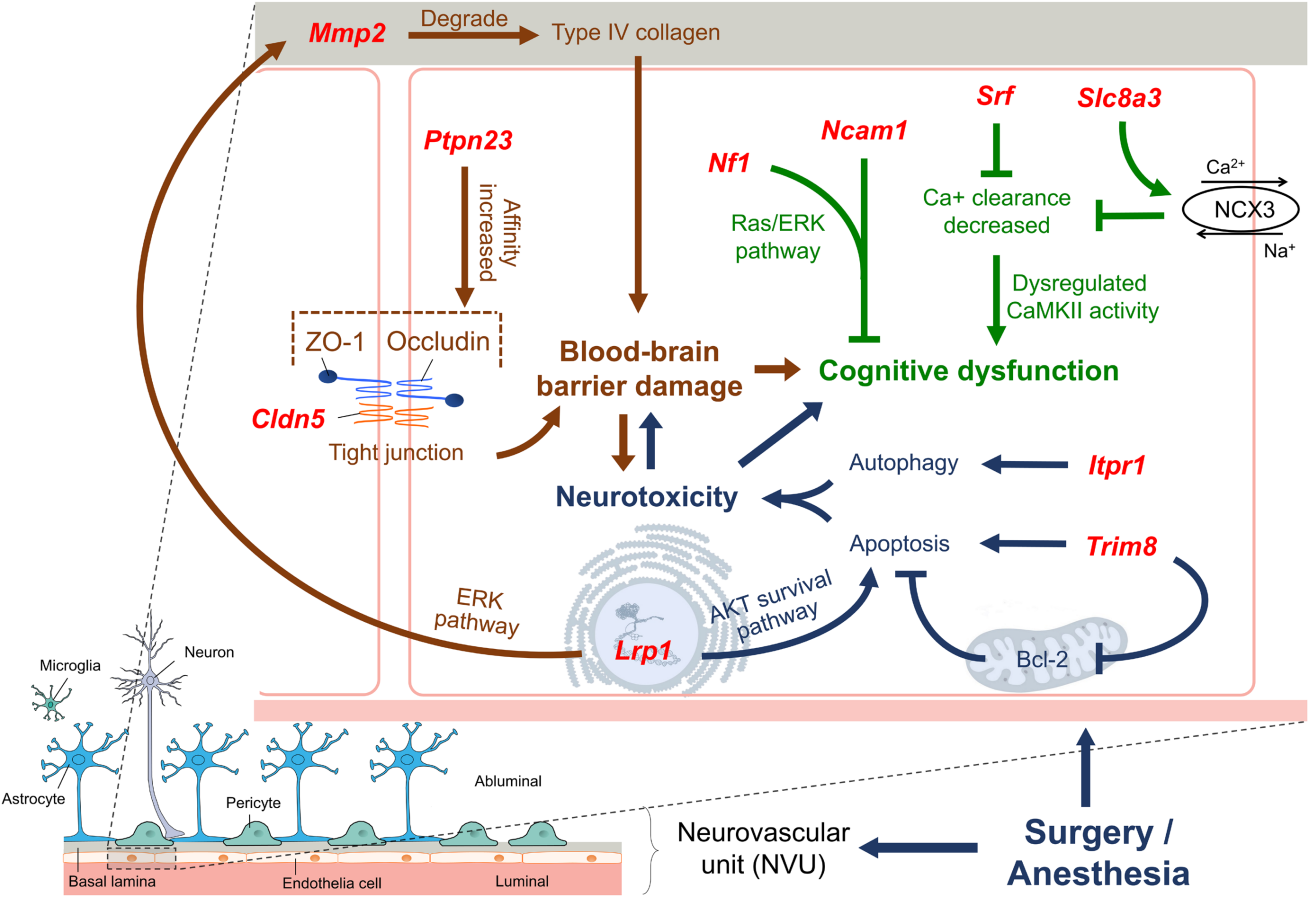
Genes (red) and hypothetical mechanisms/signaling pathways related to neurotoxicity (blue), blood–brain barrier (brown) and cognitive function (green) during perioperative period

**FIGURE 12:**
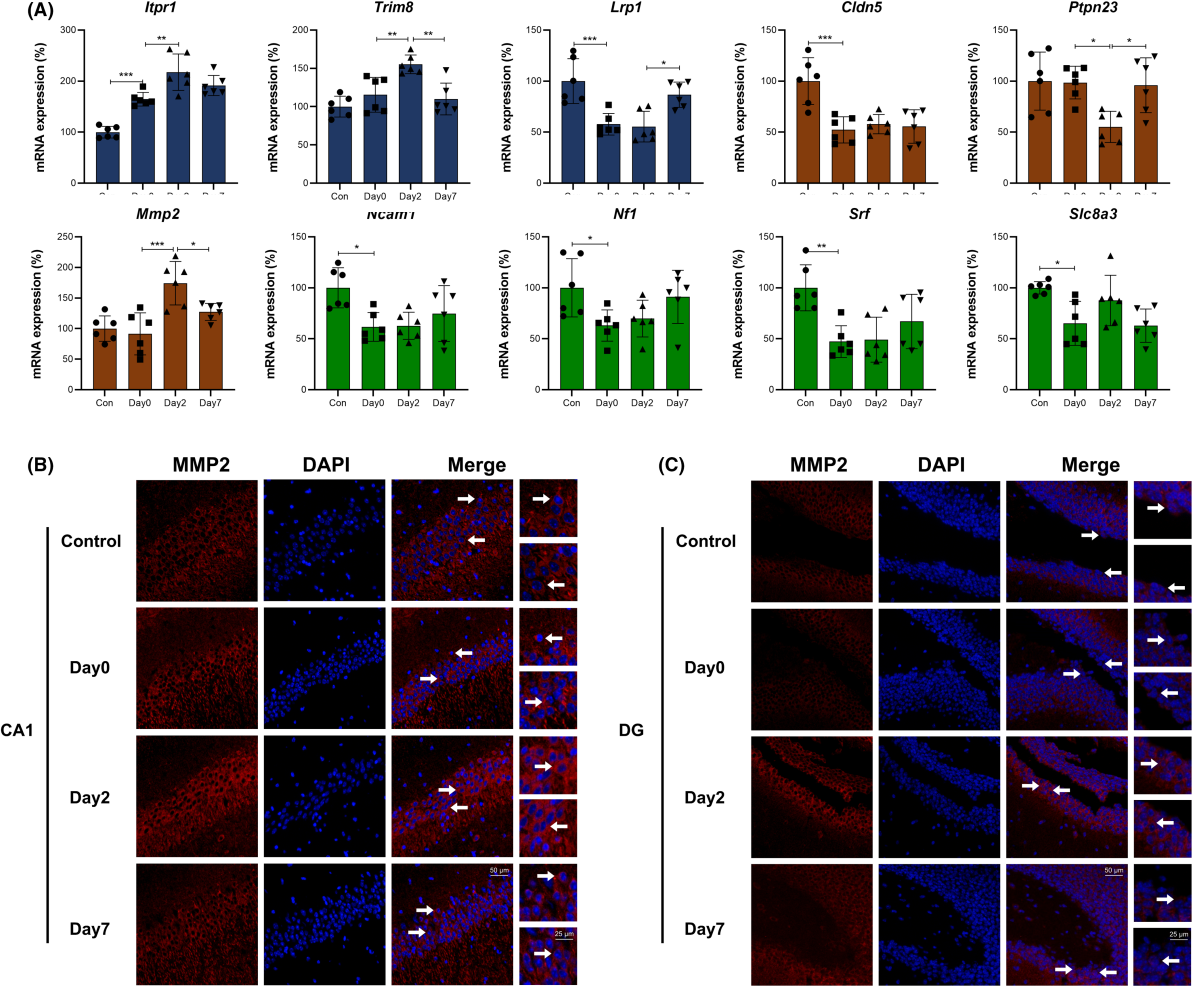
(A) qPCR verification for genes in Figure 11. The columns show gene expressions of Con, Day0, Day2, and Day7 groups (Means ± SD, *p < 0.05, **p < 0.01 and ***p < 0.001). (B and C) Immunofluorescence images show MMP2 (CY3, red) in CA1 region and DG of hippocampus. DAPI shows the nucleus in blue. Arrows point to the typical MMP2 distribution, which are provided as high magnification images on the right. Magnification 200× and 400×, scale bar 100 and 25 μm

Based on GO functional annotation and enrichment analysis, Neuronal alteration, Synapse alteration, Neurotransmitter, Biological rhythm, Oxidative stress, Metabolism, Neurotoxicity, Blood–brain barrier and Cognitive function were classified into 3 modules, then 30 DEGs and related genes were selected based on these categories. The first module included 4 genes in Neuronal alteration (*Ezh2*, *Klf4*, *Tsc2*, *Mtor*), 3 in Synapse alteration (*Rims1*, *Arf6*, *Map1a*) and 4 in Neurotransmitter (*Htr1a*, *Htr1b*, *Dvl3*, *Celsr1*; Figures 7 and 8). The second module included 3 genes in Biological rhythm (*Cry2*, *Fbxl3*, *Fbxl21*), 3 in Oxidative stress (*Itpr1*, *Micu1*, *Ppara*) and 3 in Metabolism (*Mylip*, *Gpld1*, *Sirt1*; Figures 9 and 10). The third module included 3 genes in Neurotoxicity (*Itpr1*, *Trim8*, *Lrp1*), 4 in Blood–brain barrier (*Ptpn23*, *Claudin5*, *Plectin*, *Mmp*2) and 4 in Cognitive function (*Slc8a3*, *Srf*, *Nf1*, *Ncam1*; Figures 11 and 12). qPCR verifications were performed for the abovementioned 30 genes (*n* = 6). Considered their functions and the results, 27 genes, except for 3 genes (*Tsc2*, *Dvl3*, *Plectin*), have been used to construct the potential modulation network.

Figures 7 and 8 showed Neuronal alteration, Synapse alteration and Neurotransmitter related genes and mechanisms/signaling pathways. qPCR verification showed that *Rims1*, *Htr1a*, *Htr1b* and *Arf6* were downregulated in intraoperative period, and maintained this level in early and late postoperative periods. *Celsr1* and *Mtor* were downregulated in intraoperative period, and went up in early and late postoperative periods, respectively. *Map1a* was downregulated in early postoperative period and went up in late postoperative period. *Klf4* was upregulated in intraoperative period and downregulated in early and late postoperative periods (Figure 8A). The fluctuant *Htr1a*/*b* expression of synapse leads to neurotransmitter dysregulation. *Celsr1* plays vital role in synaptic assembly and neurotransmitter release through Wnt pathway.^23^ These genes and mechanisms lead to abnormal neurotransmitter release. *Rims1* and presynaptic cytoskeleton guide synaptic vesicles to active zone and accelerate vesicle fusion. *Arf6* is related to dynamic vesicle recycling to maintain the structural and functional integrity of presynaptic terminals.^24^ *Map1a* participates in synaptic plasticity and contributes to the formation of neural circuits.^25^ These genes/mechanisms and abovementioned neurotransmitter dysregulation affect perioperative synapse alteration. EZH2 (encoded by *Ezh2*), a functional enzymatic component of PRC2 complex, affects H3K27me3 and SOCS3 level, then causes TLR‐induced NF‐κB activation and inflammatory gene expressions. *Klf4* acts as a binding partner of pNF‐κB, and co‐operatively upregulates inflammatory cytokines and neuroinflammation. Dysregulated PI3K/AKT pathway and *Mtor* affect the neural network remodeling through axonal myelination. These genes/mechanisms and synapse alteration lead to perioperative neuronal alteration (Figure 7). Typical DEG expressions were further investigated through immunofluorescence. Considered the role of ARF6 (encoded by *Arf6*) in synaptic and neuronal alteration, it was selected for verification with immunofluorescence. Due to previous studies, the hippocampal CA1 region is related to long‐term potentiation and encoding of synaptic memory,^26^ and dentate gyrus (DG) serves as an important role in engram maintenance and remote memory generalization.^27^ Thus, these 2 regions were chosen as the investigation targets. The results showed that ARF6 mainly existed in the cytoplasm of pyramidal cells in CA1 and granular cells in DG, and perioperative expression of ARF6 in both regions were showed in Figure 8B,C.

Figures 9 and 10 showed Biological rhythm, Oxidative stress and Metabolism related genes and mechanisms/signaling pathways. qPCR verification showed that *Gpld1*, *Ppara* and *Fbxl3* were downregulated in intraoperative period, and maintained this level in early and late postoperative periods. *Cry2* and *Fbxl21* were downregulated in early postoperative period and went up instantly in late postoperative period. *Mylip* was downregulated in intraoperative period and went up in early postoperative period. *Sirt1* was downregulated in intraoperative period, maintained this level in early postoperative period and recovered to baseline in late postoperative period. *Itpr1* was upregulated in both intraoperative and early postoperative periods. *Micu1* was downregulated in intraoperative and late postoperative periods, and there was no significant change in early postoperative period (Figure 10A). GPLD1 (encoded by *Gpld1*) is a glycosylphosphatidylinositol (GPI) degrading enzyme that hydrolyzes the inositol phosphate linkage and releases GPI‐anchored proteins. *Mylip* ubiquinates and degrades low‐density lipoprotein (LDL) receptor, which causes LDL accumulation in plasma. *Sirt1* affects the function of insulin receptor substrate 1 (IRS1), then alters insulin sensitivity and causes resistance. These genes / mechanisms lead to metabolic disorder. *Sirt1* facilitates the deacetylation of FOXO1α and PGC1α,^28^ then affects catalase level, mitochondrial biogenesis and oxidative stress. *Sirt1* and PI3K/AKT pathway also activate *Itpr1* and mediate endoplasmic reticulum (ER) calcium release. *Micu1* promotes the calcium flow into mitochondria, which causes ROS generation, as well as mitochondrial membrane potential (MMP) and ATP decrease.^29^ *Ppara* mediates autophagy related processes in neurons^30^ and promotes the neurotrophic factor (BDNF) production,^31^ consequently reduces the Aβ deposition. These genes/mechanisms lead to oxidative stress, which further leads to metabolic disorder. *Cry2* exerts influence on biological rhythm, and its expression is affected by CLOCK‐BMAL1‐E‐box feedback loop. *Fbxl21* stabilizes CRY2 and *Fbxl3* degrades CRY2, respectively. The decreased ATP supply also affects CRY2 through ubiquitination process.^32^ These mechanisms and metabolic disorders lead to perioperative fluctuation of biological rhythm (Figure 9). The immunofluorescence verification showed that SIRT1 existed in both nucleus and cytoplasm of pyramidal cells in CA1 and granular cells in DG, and perioperative expression of SIRT1 in both regions were showed in Figure 10B,C.

Figures 11 and 12 showed Neurotoxicity, Blood–brain barrier and Cognitive function related genes and mechanisms / signaling pathways. qPCR verification showed that *Cldn5*, *Ncam1*, *Nf1*, *Srf* and *Slc8a3* were downregulated in intraoperative period, and maintained this level in early and late postoperative periods. *Trim8* and *Mmp2* were upregulated in early postoperative period and went down in late postoperative period, while *Ptpn23* was downregulated in early postoperative period and went up in late postoperative period. *Lrp1* was downregulated in intraoperative period, maintained this level in early postoperative period and recovered to baseline in late postoperative period (Figure 12A). *Itpr1* mediates autophagy under cellular stress. *Trim8* facilitates apoptosis through Bcl‐2 inhibition,^33^ and *Lrp1* attenuates apoptosis through AKT survival pathway. These genes / mechanisms lead to neurotoxicity. Claudin‐5 (encoded by *Cldn5*), Occludin and ZO‐1 play vital roles in tight junction formation. *Ptpn23* dephosphorylates Occludin and increases its affinity with ZO‐1.^34^ *Lrp1* promotes *Mmp2* expression through ERK pathway, then the high expressed *Mmp2* degrades type IV collagen and affects tight junction as well as basal lamina. These genes / mechanisms lead to BBB damage, and there are interactions between neurotoxicity and BBB damage. *Nf1* is a Ras/ERK pathway suppressor and neuroprotective factor. *Ncam1* is important for long‐term memory formation. *Srf* and *Slc8a3* affect the encoding of sodium‐calcium exchanger NCX3, and regulate calcium outflow. These genes / mechanisms and abovementioned mechanisms lead to cognitive dysfunction. The immunofluorescence verification showed that MMP2 mainly existed in cytoplasm of pyramidal cells in CA1 and granular cells in DG, and perioperative expression of SIRT1 in both regions were showed in Figure 12B,C.

**FIGURE 13:**
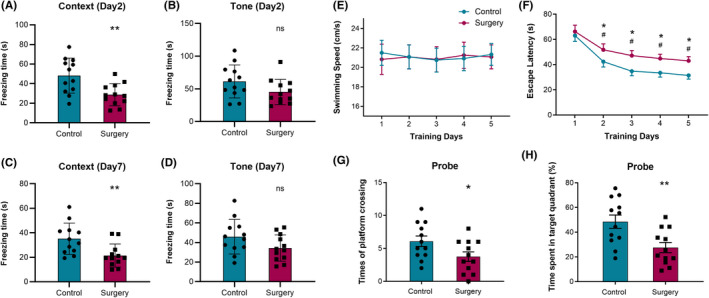
Fear conditioning test showed that in the context test, compared with control group, the freezing time decreased significantly at 2 days (A) and 7 days (C) in surgery group. In the tone test, the freezing time did not decrease significantly at 2 days (B) or 7 days (D) in surgery group (*n* = 12 for both groups). Morris water maze test showed that in the place navigation test, the swimming speed did not change significantly in both groups (E), but the escape latency increased significantly after surgery (F). In the probe test, both the times of platform crossing (G) and the time spent in target quadrant (H) decreased significantly after surgery. **p < 0.01, *p < 0.05, ns: not significant

Behavior tests proved the occurrence of hippocampus‐dependent cognitive dysfunction during perioperative period. 24 aged mice were subjected to FCT and divided into control and surgery groups (*n* = 12). In the context test, the freezing time decreased significantly at 2 days after surgery (48.17 ± 17.88 vs. 28.61 ± 11.26, *p* = 0.0041, Figure 13A) and 7 days after surgery (35.14 ± 12.74 vs. 21.45 ± 9.461, *p* = 0.0068, Figure 13C) in the surgery group. In the tone test, there was no significant between two groups at 2 days after surgery (61.27 ± 25.22 vs. 45.21 ± 19.29, *p* = 0.0938, Figure 13B) or 7 days after surgery (45.90 ± 17.72 vs. 34.33 ± 13.44, *p* = 0.0852, Figure 13D). Another 24 mice were subjected to Morris water maze test and divided into control and surgery groups (*n* = 12). The place navigation test began at 1 day after surgery, and during 5 training days, the swimming speed maintained constant and showed no significance between two groups (Figure 13E), the escape latency decreased significantly as the training went on and this trend appeared less pronounced in surgery group (Figure 13F). In the probe test (1 day after last training), the times of platform crossing and the time spent in target quadrant decreased significantly in surgery group (6.08 ± 0.79 vs. 3.75 ± 0.72, *p* = 0.0402, 48.52 ± 5.41 vs. 27.59 ± 4.13, *p* = 0.0055, Figure 13G,H). These results suggested the occurrence of POCD in aged mice. The cognitive dysfunction is hippocampus‐dependent, and the perioperative gene expression changes and related mechanisms / modulation networks in the hippocampus could be its foundation.

## 4 DISCUSSION

In the present study, we analyzed the perioperative gene expressions in aged hippocampus and figured out their patterns in 3 periods. These periods could cover the major perioperative pathological changes. For example, inflammatory factors could be detectable in the circulation within 30 min after surgery which influences neuroimmune circuits,^7^ disrupted neuroglial metabolic coupling occurs in 1–3 days after surgery,^35^ and hippocampal lipid peroxidation occurs 7 days after surgery.^8^ Here, 328, 3597 and 4179 DEGs were screened out in intraoperative period, early and late postoperative period. Upregulated genes accounted for 61.9%, 44.2% and 51.4% of all DEGs in these periods. The major BP terms were divided into 9 categories including Neurotransmitter, Synapse alteration, Neuronal alteration, Metabolism, Oxidative stress, Biological rhythm, Blood–brain barrier, Neurotoxicity and Cognitive function. The negative and positive regulation terms, top DEGs and TFs involved in these categories were summarized. To better analyze the involved mechanisms and pathways, we divided the categories into 3 modules, the DEG intersections, signaling pathways and modulation networks of these modules were summarized and constructed, respectively.

Previous studies have indicated perioperative alterations in neuronal survival (neuroapoptosis) and structures (altered dendritic and glial morphology),^36^ our results further revealed the possible alteration directions. In intraoperative period, the major pathological processes were BBB and neuronal alteration. Their directions were both positive alterations, which bring early influences for the occurrence of PND. In postoperative periods, the major pathological processes were synapse and neuronal alterations, metabolic disorder, oxidative stress, BBB damage and neurotoxicity. Negative alterations existed in neurotransmitter, synapse and neuronal alterations in early postoperative period. They shifted from negative to positive alterations in late postoperative period, which indicated partial improvements. Positive alterations were the major directions for other processes, and for metabolic process, the alterations were more obvious in late postoperative period. The results also showed the involvement of TFs during perioperative period, and the top 10 TFs were *Klf4*, *Hbp1*, *Srf*, *Zeb2*, *Egr1*, etc. As previous studies revealed, *Klf4* could regulate cell survival progress.^37^ *Hbp1* contributes to the pro‐inflammatory macrophage/microglia‐mediated response.^38^ *Srf* mediates the synaptic activity, and controls the neuronal outgrowth.^39^ *Zeb2* is a key developmental regulator of CNS,^40^ and *Egr1* is necessary for long‐term potentiation and memory consolidation.^41^ Therefore, these TFs could play important roles in the occurrence of PND.

As the results indicated, abnormal neurotransmitter release, neuronal and synapse alterations emerged in the hippocampus during perioperative period. Neuroinflammation occurring in neurons and microglia is a key feature of PND. In these cells, NF‐κB is activated via toll‐like receptors and promotes the production of inflammatory cytokines including IL‐1β, IL‐6 and TNF‐α.^42^ *Klf4* and *Ezh2* exert vital roles in this inflammatory signaling pathway.^43^, ^44^ Our previous study indicated that altered intestinal microbiota after surgery‐induced intestinal inflammation, affected the integrity of intestinal barrier and BBB,^16^ which could be an important cause of neuroinflammation. PI3K/AKT/mTOR pathway affects axonal myelination,^45^ and influence neural networks. During perioperative period, mitochondrial fission and fusion dynamics are disturbed,^46^ which leads to the decrease of mitochondrial transmembrane potential and ATP production, and then affects synaptic plasticity through dendritic remodeling. Vesicle transmission and trafficking in terminals influence synaptic function, and the interaction of *Rims1* and *Rab3a* is necessary for the process.^47^ Some vesicle recycling‐associated proteins (such as ARF6) are responsible for several neurologic and psychiatric diseases including Schizophrenia.^48^ Therefore, surgery‐related abnormal neurotransmitter and synapse alteration influence the information flow between neurons, which are crucial mechanisms for PND and other perioperative diseases.

Metabolic disorder, oxidative stress and biological rhythm alteration emerged in the hippocampus during perioperative period. Surgery and anesthesia‐related metabolic disorder occurring in neurons and glia involves multiple aspects including lipid, protein, carbohydrate, etc. Our study showed that perioperative lipid metabolic disorder in aged hippocampus was related to transcription factor SREBP1c.^17^ The present results indicated differential expression of cholesterol regulator *Mylip*. *Mylip* affected cholesterol level via LXR/MYLIP/LDLR pathway,^49^ caused hypercholesterolemia, and was a fundamental cause for PND.^50^ *Sirt1* affects insulin reactivity via PTPN1, IRS and AKT pathways.^28^ Insulin resistance is implicated in AD,^51^ and could also be the mechanism for PND. Surgery‐related Ca^2+^ overload in mitochondria is due to mitochondrial calcium uniporter (MCU, encoded by *Micu1*). *Itpr1* could provide flow source by releasing calcium from ER, and *Sirt1* could change acetylation levels of MCU and affect the calcium flow.^52^ Previous study showed that both Ca^2+^ efflux via *Itpr1* and Ca^2+^ influx via MCU promoted oxidative stress,^53^ and led to membrane permeability increase, cytochrome c release, respiratory inhibition in neurons.^54^ *Ppara* also affects the process through regulating Aβ deposition.^30^ Biological rhythm disorder is the hallmark for aging and neurodegenerative diseases.^55^ Our results revealed expression changes of rhythm‐related genes, such as *Cry2*, *Fbxl3* and *Fbxl21*,^19^, ^56^ which indicate biological rhythm alteration during perioperative period, and could be another mechanism for PND.

Neurotoxicity, BBB damage and cognitive dysfunction also emerged in the hippocampus during perioperative period. Surgery and anesthesia‐related neurotoxicity includes apoptosis and autophagy, which affect the function of neurovascular unit and neurons. Apoptosis could be triggered by DNA damage, including DNA double‐strand breaks and oxidative DNA adducts. P53‐mediated pathway and PI3K/AKT pathway are involved in the process.^57^, ^58^ Autophagy could be activated in a perioperative mTOR/IP3R‐dependent manner.^59^ Sustained autophagy induced neuron death through protein recycling process impairment and critical cellular constituent depletion.^60^ Surgery‐induced BBB damage is also an important perioperative pathological characteristic. BBB damage allows the entry of neurotoxic debris, cells and pathogens, which is critical for CNS inflammation and immune responses.^7^ The disruptions of tight junction and basement membrane are the key mechanisms for this process, which involves Claudin 5, ZO‐1 and MMPs.^61^ Fluid flow also plays roles in BBB regulation and endothelial glycocalyx‐related gene expressions.^62^ Platelet‐derived growth factor B is critical for pericyte coverage and BBB function,^63^ while cerebral hypoperfusion, BBB disruption and CSF Aβ decrease are related to long‐term neurological deficits.^64^ Just as modulation networks hint, POCD is caused by joint action of multiple CNS cell types under surgical stress. The symptoms of POCD include impairments of memory, attention, action and perception, and several DEGs found in the present study (*Ncam1*, *Srf*, etc.) were related with cognitive function.^65^ The present GO analysis revealed changes of learning and long‐term memory, and KEGG analysis revealed pathways related to neurodegenerative diseases including AD, Parkinson disease and Huntington disease.

## 5 CONCLUSION

In the present study, we identified gene expression patterns in the aged hippocampus during different perioperative periods, and summarized the major involved processes including Neurotransmitter, Synapse alteration, Neuronal alteration, Metabolism, Oxidative stress, Biological rhythm, Blood–brain barrier, Neurotoxicity and Cognitive function. Then we constructed potential signaling pathways and modulation networks in these pathological processes. The results provide insights into overall mechanisms during perioperative period for PND and reveal the potential therapeutic gene targets, which are valuable for the prevention and treatment of perioperative CNS disorders from genetic level in the future.

## AUTHOR CONTRIBUTIONS

ZS performed the experiments, analyzed the data and wrote the manuscript. JY contributed to experiments, data analysis and manuscript revision. BZ and ML contributed to data analysis and manuscript revision. YQ contributed to animal experiments and data analysis. WX contributed to data analysis. TX and HZ contributed to the project supervision and manuscript revision. CN designed the project, supervised the experiments, drafted and revised the manuscript.

## CONFLICT OF INTEREST

The authors declare no financial or commercial conflict of interest.

## Data Availability

The datasets generated for this study can be found in the Dryad Digital Repository (https://doi.org/10.5061/dryad.jsxksn0cj).
